# Involving Health Professionals in the Development of Quality and Safety Dashboards: Qualitative Study

**DOI:** 10.2196/42649

**Published:** 2023-06-12

**Authors:** Frank Christian van de Baan, Stijn Lambregts, Esther Bergman, Jasper Most, Daan Westra

**Affiliations:** 1 Department of Health Services Research Care and Public Health Research Institute Maastricht University Maastricht Netherlands; 2 Department of Quality, Safety and Quality Improvement Zuyderland Medical Centre Heerlen Netherlands; 3 Department of Research Zuyderland Academy Zuyderland Medical Centre Sittard-Geleen Netherlands; 4 Department of Orthopaedics Zuyderland Medical Centre Sittard-Geleen Netherlands

**Keywords:** quality improvement, dashboard, user involvement, innovation, health care provider, health care professional, feedback, opinion, perspective, qualitative, constant comparative method

## Abstract

**Background:**

Dashboards are an important tool for hospitals to improve quality and safety performance. However, implementing quality and safety dashboards often does not increase performance due to a lack of use by health professionals. Including health professionals in the development process of quality and safety dashboards can improve their use in practice. Yet, it remains unclear how a development process involving health professionals can be executed successfully.

**Objective:**

The aim of this study is twofold: (1) to delineate how a process whereby health professionals are included in the development of quality and safety dashboards can be facilitated and (2) to identify the factors that are important to consider in order to make that process successful.

**Methods:**

We conducted a qualitative, in-depth exploratory case study in which we analyzed 150 pages of internal documents and interviewed 13 staff members regarding the development of quality and safety dashboards within 2 care pathways of a hospital that has experience in such development. The data were analyzed inductively using the constant comparative method.

**Results:**

We found that the development of quality and safety dashboards in collaboration with health professionals was facilitated through a five-stage process: (1) familiarizing participants with dashboards and the development process; (2) brainstorming about potential indicators to be included in the dashboard; (3) prioritizing, defining, and selecting indicators to be included in the dashboard; (4) examining how the indicators can be visualized; and (5) implementing the dashboard and following up on its use. To enhance the success of the process, 3 factors were deemed important. The first is to create and maintain broad involvement, ensuring that various professions are represented and take ownership of the dashboard. Here, potential barriers include gaining engagement from peers not directly involved in the process and maintaining involvement after the initial implementation of the dashboard. Second, unburdening, whereby quality and safety staff facilitate a structured process that has little additional burden for professionals. For this, time management and a lack of collaboration with departments responsible for delivering the data might be an issue. Lastly, focusing on relevance for health professionals, which refers to the inclusion of indicators with value for health professionals. For this factor, a lack of consensus on how indicators should be defined and registered might be a barrier.

**Conclusions:**

Health care organizations seeking to develop quality and safety dashboards in collaboration with health professionals can use a 5-stage process. To enhance the success of the process, organizations are advised to focus on 3 key factors. For each of the key factors, potential barriers should be taken into account. Engaging in this process and attaining the key factors could increase the likelihood that the dashboards are used in practice.

## Introduction

Improving the quality and safety of care is a core focus of hospitals around the world [1-4]. To do so, hospitals identify and monitor relevant quality and safety indicators [5-7]. Yet, as the number of indicators and amount of available data expands, effective day-to-day monitoring and improvement become increasingly difficult [8]. Quality and safety dashboards are therefore becoming an increasingly popular tool to create a clear overview of various indicators and the progress made [9-12]. However, reviews show that the success of these dashboards is far from guaranteed and that the lack of improved outcomes typically stems from either a lack of use or improper use of the dashboard by health professionals [10,11].

Traditionally, quality and safety dashboards have been developed without the involvement of health professionals [13-19]. While some of these have been shown to improve quality and safety outcomes [13,14], overall the benefit of quality and safety dashboards is contested, with recent paradigms attributing the absence of improvements to a lack of inclusion of health professionals’ perspectives [10-12]. Studies have shown that without the inclusion of health professionals during development, these dashboards can lack usefulness (eg, display content that is perceived as irrelevant and unreliable by the user) and usability (eg, visualize data in an incomprehensible way) [10,11,20]. Moreover, enforcing the use of dashboards in a “top-down” manner can interfere with professionals’ autonomy and routines, making dashboards a burden to users [12,21]. In such instances, health professionals tend to create workarounds that minimize the perceived negative impact of the dashboard (such as additional administrative burden), use the technology incorrectly, or simply disregard it [22]. Fortunately, a recent study demonstrated the development of a patient experience dashboard whereby clinical and nonclinical staff were included in the development process [23]. However, while the study provides valuable insight into the type of content derived when health professionals are included, how the process itself can best be facilitated remains unclear.

Overall, the utility of quality and safety dashboards is hampered by a lack of health professional engagement in the development process. While studies highlight the importance of including health professionals in the process, it remains unclear how the process of developing quality and safety dashboards in collaboration with health professionals can be executed successfully. The aim of this study is twofold: (1) to delineate how a process whereby health professionals are included in the development of quality and safety dashboards can be facilitated and (2) to identify the factors that are important to consider in order to make that process successful. The results of this study can provide hospitals with insight into the aspects to focus on when engaging in the process of developing quality and safety dashboards together with health care professionals and can bridge the gap between the promise and actual usage of quality and safety dashboards. As such, the study offers an important contribution to realizing quality and safety improvements in hospitals.

## Methods

### Overview

We conducted an in-depth exploratory case study, interviewing staff from 2 care pathways of a large teaching hospital with experience in developing quality and safety dashboards together with health professionals. In line with the literature, we define care pathways as patient-focused care provided by multidisciplinary teams to a well-defined group of patients in a well-defined time period [24]. Furthermore, within this study, dashboards are defined as digital information delivery systems that provide an overview of quality and safety indicators within a specified timeframe [25,26]. This study followed the COREQ (Consolidated Criteria for Reporting Qualitative Research) [27].

### Setting

With more than 206,000 unique patient visits, 980 beds, and over 4500 full-time equivalent staff in 2019, the hospital included in this study is one of the largest hospitals in the Netherlands [28]. The hospital has been engaged in the process of developing and implementing quality and safety dashboards together with health professionals since 2019. So far, this process was undertaken within 13 care pathways. While the exact composition differed, the development teams generally consisted of medical specialists, nurses, and team leaders, always guided by a member of the quality and safety department, with care pathway managers receiving updates throughout the process. During the development process, minutes, presentations, and reports were created by the quality and safety supervisors, to which participants could respond via email.

### Data Collection and Sample

For this study, we collected data from the hip fracture care pathway and the maternal care pathway. We selected these care pathways since they were among the first to have fully completed the entire development process. Furthermore, for these care pathways, extensive minutes and other types of documents, such as email responses by participants and presentations, were available. The fact that both pathways had already completed the process allowed participants to reflect on any challenges that persisted after the implementation of the dashboard.

The hip fracture care pathway is a multidisciplinary care pathway in which staff from orthopedics, surgery, and geriatrics work together. The care pathway revolves around care for elderly patients who have broken their hips. This care pathway was created in 2018 as a means to optimize the flow of hip fracture patients to nursing homes. The quality and safety dashboard covers the entire care path, from admission to discharge. It is available to all trauma surgeons and nurses and can be accessed via the internal website of the hospital.

The maternal care pathway focuses on safe and healthy childbirth. This care pathway includes staff from gynecology, obstetrics, and pediatrics. This care pathway was created in 2015 in light of the concentration of all childbirths from 2 hospital locations to one central location. As with the hip fracture care pathway, the quality and safety dashboard used covers the entire care path. It is available to all gynecologists, pediatricians, obstetricians, and nurses and can be accessed via the internal website of the hospital.

Regarding the data, we collected documents from and conducted interviews with members of both care pathways. We were granted access to the documents on the development processes in both care pathways through author SL, who is a member of the hospital’s quality and safety department. In total, we collected 150 pages of documents. The use of documents allowed for data triangulation, whereby the documents both served as a means to inform the interview guide and as an additional source of data for the findings. Fifteen individuals were involved in the development process, and all were subsequently invited for an interview by the first author via email. The email contained an information letter and informed consent form, providing details about the project, interviews, and ethical considerations, such as the storage and use of gathered interview data. Of all invited individuals, 13 individuals participated in an interview (ie, 2 quality and safety supervisors, 4 managers, 4 medical specialists, and 3 nurses), a sufficient sample size for data saturation for this type of study [29]. Due to a high workload as a result of the COVID-19 pandemic, 2 individuals could not participate. As can be seen in Table 1, there was a relatively equal distribution among the interviewees in terms of sex, function, and years of tenure at the hospital.

**Table 1:** Interviewee characteristics.

			Hip fracture care pathway (n=7), n		Maternal care pathway (n=6), n
**Sex**					
	Male	3		3	
	Female	4		3	
**Function**					
	Quality and safety employee	1		1	
	Manager	2		2	
	Medical specialist	2		2	
	Nurse	2		1	
**Years of tenure at hospital**					
	2-5	2		1	
	6-10	3		1	
	11-15	1		1	
	16-20	1		N/A^a^	
	>20	N/A		3	

^a^N/A: not applicable.

Interviews were conducted by the lead author, an experienced qualitative researcher, in Dutch via Zoom due to COVID-19 restrictions and took place between December 2021 and March 2022. The interviews lasted, on average, 30 minutes. The topic list guiding the interviews was created based on literature and the retrieved documents (Multimedia Appendix 1). The main focus of the interviews was to understand the approach to the development process and the experience of staff with that process. To get a full understanding of the approach used during the development process, the quality and safety employees supervising the process were interviewed first. The 2 interviewees were asked to reflect on the preparations of the different sessions and process as a whole, the content of and their experience with the sessions, points that went well or should be improved, and general lessons learned. The remaining respondents were interviewed thereafter. For these interviews, the interview guide was slightly changed based on the first 2 interviews, whereby it focused less on preparation and content but more extensively on experiences and lessons learned (Multimedia Appendix 1).

### Ethical Considerations

This study was granted ethical approval by the Faculty of Health, Medicine and Life Sciences Research Ethics Committee of Maastricht University under reference number FHML-REC/2022/002. Approval for collecting and analyzing the written documents was obtained from the head of the quality and safety department. All names were taken out of the written documents by the lead author. Participation was voluntary, and interviewees did not receive compensation. All participants provided written informed consent before participating in the study. Interviews were recorded with an audio recorder and transcribed verbatim. For data protection, all audio recordings were deleted after being transcribed. Identifiable information was taken out of the transcripts by the lead author so that the data was pseudonymous. A member check was conducted by asking all interviewees to provide corrections to the summary and comments to the transcript of the interview, thereby also giving them the opportunity to ensure no information that could do them harm would be included. None of the interviewees requested that the transcript be changed. Only the lead author had access to the original audio recordings, and only the lead and last author had access to the interview transcripts.

### Analysis

Documents and transcripts were uploaded into Atlas.ti (version 9; Atlas.ti Scientific Software Development GmbH) for analysis. The first author engaged in constant critical discussion during data collection and analysis with the other authors to reduce bias [30]. The constant comparative method was used to analyze the data [31,32]. The analysis consisted of several phases. First, before conducting the interviews, the documents were inductively coded by the lead author in order to gain insight into the development process and the content of the different stages and to be able to construct the interview guide. Here, a low level of abstraction and a low degree of interpretation were used, as they regarded more concrete descriptions and did not require an understanding of the underlying meaning of the text [33]. The different codes were grouped into codes containing information on the process as a whole and codes containing information on specific stages of the process. This also allowed us to identify which aspects the interviews should focus on in support of the study’s aim. Through the analysis, for example, it became apparent that stage 2 of the process revolved around brainstorming and that a distinction was made between 4 types of indicators that could be included in a dashboard. We used this information for the findings section on the content of the different stages. The documents, however, did not specifically state whether there are important aspects to consider in order for this session to be successful. Therefore, we included a question in the interview guide asking participants to reflect on this.

In the next phase, the interview data were inductively coded by the lead author. Here, all elements relating to the process itself and participants’ views on the process were coded. As coding this type of data required higher abstraction and interpretation, the last author analyzed 3 random anonymized transcripts, and the coding was compared with the coding of the first author to increase reliability. No major differences in coding were found. Next, when coding was completed, the lead author went back to the data set to group codes into main themes, constantly going back and forth through the data, seeking similarities and differences among the many codes, and critically assessing whether codes could be grouped into common themes. During this process, emerging insights were shared and discussed with the total research group (5 researchers in total)—who could provide a more critical, outsider perspective—until the analysis was completed [30]. Through these discussions, we, for example, distilled *creating broad involvement* as an emerging theme arising from a combination of open codes relating to the broad inclusion of health professionals in the development process, such as the codes *representation of different functions* and *peer engagement*. We then ultimately distilled the main factor *creating and maintaining broad involvement* through the combination of the second order codes *creating broad involvement* and *maintaining broad involvement*, since both related to the broad involvement of health professionals (Multimedia Appendix 2).

## Results

### Overview

In light of the first study aim, our analysis revealed that the development of quality and safety dashboards in collaboration with health professionals was facilitated through a 5-stage process, beginning with a kickoff session and ending with implementation. Furthermore, with regards to the second study aim, we identified 3 factors that participants deemed important to focus on during the process to enhance success. First, creating and maintaining broad involvement was deemed essential by participants. Second, ensuring that the process was of little additional burden to participants was deemed important. Lastly, participants highlighted the importance of focusing on relevance for health professionals. Below, we further describe the 5-stage process and the 3 key factors.

### The 5-Stage Process

Based on the document analysis and interviews with the quality and safety staff members, we find that the development process of the quality and safety dashboards consisted of 5 stages (see Textbox 1). Each stage consisted of at least one session where all participants met physically. Overall, the process was scheduled to take between 3 and 4 months. Engaging in the process always began bottom-up, with staff from the care pathways approaching the quality and safety department with a request for developing a quality and safety dashboard. A team was then created of members of the care pathways who had an affinity for quality and safety, representing the different functions and departments associated with the care pathway (ie, team leaders, medical specialists, and nurses). Throughout the process, the same participants were continuously involved. The sessions were chaired by a quality and safety supervisor, who also made preparations (for example, identifying specific characteristics of the care pathway before the first session was held) and conducted follow-ups:

For the care pathway, the disease for which it is, you look into the literature. What do we have at the moment? Do we already need to report certain KPI’s [key performance indicators]? Do these need to be delivered? Which of those are already being registered? Because that’s of course our goal, no extra administrative burden.... You look at the inspection [national quality and safety inspectorate], that institute, and so on. You make preparations for that pathway before you enter session zero [the kickoff].

R2, quality and safety supervisor

**Summary of stages.**

**Stage 1: Kickoff**

Participants are familiarized with the concept and potential value of quality and safety dashboards for continuous improvement of their care pathways, as well as the concept of quality and safety indicators in general. Furthermore, the upcoming development process is being explained. In order to give participants an idea of what a dashboard entails and what the goal of the process is, participants are shown examples of dashboards previously created or those used in other settings (eg, the COVID-19 dashboard created for the public by the World Health Organization).

**Stage 2: Brainstorm**

Participants brainstorm about potential indicators to be included in the dashboard. To aid participants in thinking about indicators, they are encouraged to consult sources relevant to their field, such as inspectorate protocols, International Consortium for Health Outcome Measurements sets, medical specialists’ association guidelines, and information from patient representation groups. Overall, 4 types of indicators are distinguished: process indicators (relating to how care is being delivered, such as administering medication on time); and 3 types of outcome indicators, including patient-reported experience measures, patient-reported outcome measures, and clinical outcome measures. During the brainstorm stage, participants are encouraged to have an open mind, not yet thinking about the feasibility of improving certain indicators but instead thinking about the indicators they deem most important.

**Stage 3: Prioritize and define**

From the broad array of indicators listed, participants select around 10 of the most relevant indicators to be included in the dashboard. A selection of around 10 indicators is made, as sufficiently monitoring more indicators was deemed difficult. Participants further focus on the definition of each indicator (ie, reaching a consensus on the nominator and denominator and how indicators are registered), as staff can have different perceptions, which can hamper proper registration and measurement of performance.

**Stage 4: Visualize**

Participants decide on the layout of the dashboard as a whole and each indicator specifically. Considering that participants often have difficulty picturing a layout themselves, the quality and safety supervisors on forehand create one or more concept versions, which can then be used by the participants to tailor it to their preferences. While distinct, each dashboard uses the same base, including the filters on top, the core data underneath, and the indicators under the core data. The dashboard is designed in such a way that it is possible to determine statistics at the individual level using a drill-down function.

**Stage 5: Implement**

During the final session, the finalized dashboard is presented, and participants are shown how to use it. Moreover, implementation of the dashboard is discussed, specifically focusing on how its use will be embedded in the department. This includes focusing on who has access to the dashboard, the frequency of consultation, how improvements can be identified, and when and with whom the results will be discussed. Being a continuous process, participants are asked to first make use of the dashboard for 2-3 months before quality and safety supervisors consult with them on their experiences so far.

The supervisors also had contact with the business intelligence (BI) and information technology (IT) departments, which aided in designing the dashboard and collecting the data. Overall, the development process can be considered an iterative process that includes going back and forth between stages and splitting up stages, if necessary (eg, in case of lack of time or little progress made) and consisting of preparations and follow-up (via email) before and after sessions.

### The 3 Key Factors

#### Creating and Maintaining Broad Involvement

In light of enhancing a successful process, 3 factors emerged from the interview data as deemed important by participants. First, creating and maintaining broad involvement was seen by participants as an important base throughout the development process (see Table 2). Interviewees were enthusiastic about the involvement of staff from the different functions of the care pathway (ie, team leaders, medical specialists, and nurses from various departments), whereby especially those with an affinity for quality of care were included. This was said to help with a smooth process and increase the likelihood of ending up with the most relevant indicators:

What went well is that there was a group that had affinity with it. Affinity with quality of care as a whole and who also liked to think about: how do we quantify quality of care? In my opinion, that was the essential condition for arriving at any sensible quality parameters in the first place: that you are working with a group that likes to provide input.

R4, medical specialist

Having broad involvement further contributed to having a support base among all staff of the care pathway as people felt represented. To further enhance the feeling of ownership and connectedness, after each session, participants were asked to report back to and consult with their peers. This was said to increase the relevancy of the dashboard for the broader workforce as input could be provided by them. Nonetheless, from both care pathways, some interviewees mentioned that, as the process moved through the different stages, it was difficult to achieve true engagement from peers. This was said to be because peers lacked awareness of the process and end product and the influence they could exert on the process:

From every group there was one representative. That was a strength, but also a relative weakness. Because in the pathway there’s a lot of people and I had to also inform my peers. Those peers don’t feel the same as what I see and feel during the session, and what we’re doing there. So then you have to tell your peers without the slides that we’re going to make a quality dashboard. I did show them that presentation and also asked if these are the indicators that we should work with. But you notice that those sitting in the front, so those given the mandate to participate in the sessions by the peers, feel much more involved than those peers.

R5, medical specialist

To overcome the issue of achieving true engagement from peers, some suggested that quality and safety supervisors better convey to participants that they should frequently consult with their peers, asking for their input.

Maintaining involvement after the initial implementation of the dashboard in practice was said to be challenging as well. Some stated that using the dashboard frequently and discussing progress got deprioritized due to day-to-day routines. They, therefore, suggested incorporating a routine evaluation of the dashboard during monthly team meetings to maintain usage:

Because of busy days, but also absenteeism which increases workload it’s difficult to find a right moment to go further.... We do try to get it right. This afternoon, for example, we’ll sit together with the KPI workgroup. So that I plan so that we maintain focus and can see what we need.

R12, nurse

**Table 2:** Key factors for a successful development process.

Key factors and definitions	Potential barriers	Proposed operational solutions
Creating and maintaining broad involvement: Ensuring that various professions (ie, nurses, medical specialists and managers) are represented in the process and take ownership of the dashboard	Fully gaining engagement from peers who are not directly included in the process: *You notice that the nurse are not really engaged.... So you need to update the department, sit together with them, structurally asking for feedback.* [R3, nurse]	Select eager participants and remind them to frequently consult with their peers, update them on the process and ask for input: *I have a very* *enthusiastic* *nurse who also really knows a lot about it. And she really motivates and informs the rest of the team.* [R12, nurse]
	Maintaining involvement in the use of the dashboard after initial implementation: *The process [of using the dashboard] can be improved so that we also get better outcomes.... It remains a challenge for the department to really get the whole team involved.* [R12, nurse]	Incorporate routine evaluations of quality and safety performance based on dashboard data: *You need to structurally plan it [evaluation of the dashboard] as a standard part of your meetings. Because it you don’t do that you’ll have a quality dashboard which no one uses.* [R5, medical specialist]
Unburdening: Quality and safety staff facilitating a structured process that has little additional burden for participants	Time management, the process taking too long causing a lack of progress: *You need to be wary of it on forehand [to not take too long], when you know you start with it, that you agree on a timeframe and schedule sessions... We had the initial dashboard rather quickly if I remember correctly, but after that it took quite a while to continue working on it.* [R7, Manager]	On forehand plan the different sessions and be strict on moving the process forward: *What also went well... the project was well-managed. He [the quality and safety supervisor] took the lead to have periodic meetings and keep everyone on their toes to provide input. So it that sense there was some strictness.* [R4, Medical specialist]
	Lack of collaboration with the IT and BI^a^ departments: *IT support is always a point of attention for us. Often I find that a bottleneck in these types of processes of getting things digital. I find that difficult sometimes because there is sometimes unnecessary delay and then you sometimes also notice frustration.* [R9, Manager]	Good communication with the IT and BI departments, engage in expectation setting: *And Business Intelligence also indicated at the time: yes, we can continue dealing with those data requests, but what does the process look like? ...Basically the ones we asked for data were a bit overstretched. So they had something like: where is it coming from? What’s the background? And of course we had to include the BI team in that too.* [R1. Quality and safety supervisor]
Focusing on relevance for health professionals: Including quality and safety indicators in the dashboard that are of value to health professionals	Lack of proper definition and registration of indicators: *There are a number of pitfalls. On the one hand regarding the completeness of daily registration. In the beginning it was by no means always complete.... On the other hand there is quite a challenge regarding the definitions that are being used. So the moment we define length of stay—just to name one—there’s quite a lot of variability in how that is registered...and then you make wrong interpretations.* [R4, medical specialist]	Focus on reaching consensus during the development process on how indicators should be defined and registered: *What’s important, is that the data that comes out of it is reliable and correct. Because sometimes there are mistakes in that to.... In definitions or formulas or the denominator divided by the numerator or whatever. On forehand it must be clear that it is correct, before it is being presented to the staff. You need to ensure the essence is right, otherwise it will turn against you*. [R8, manager]

^a^BI: business intelligence.

#### Unburdening

Unburdening emerged as a second important factor, whereby participants experienced the process as having little additional burden. This was deemed very positive and important given the little time they had. Interviewees stated that this was the result of a well-supervised and structured process, which also allowed for a smooth transition through the different stages, ensuring that all aspects were properly covered. Interviewees further appreciated that from the beginning onward they were aware of the planning and what was expected of them, whereby after each session clear follow-ups were shared via email, including summaries of the sessions and tasks for them to be done between the sessions:

I think it was very nice that at the beginning of the sessions there was a clear plan of what the sessions would look like, that there was a clear timeline and that someone else made the arrangements for us. As someone on the work floor you’re busy anyway and if you then also have to look in the agendas and so on.... That there.... During the first meeting 6 dates were planned, so that was and advantage, because it keeps your project moving and you know for sure that everyone is available.

R5, medical specialist

The quality and safety supervisors and managers perceived it as their role to facilitate a process that meets the participants’ needs, taking away tasks that can be done by others so that participants can solely focus on the development itself. For example, this meant that the quality and safety supervisors performed all contact with the BI and IT departments, which were responsible for data delivery. As shown in Table 2, in attending to participants’ needs, the quality and safety supervisors commented that this did not mean that they were not strict if needed to keep the process moving forward, for example during the implementation stage.

Yes it [the process] shouldn’t take too long. We shouldn’t keep making adjustments. That is really something we should keep. That at some point we say: “First start using it [the dashboard] and then come back to us.”

R1, quality and safety supervisor

#### Focusing on Relevance for Health Professionals

A focus on relevance for professionals was identified as a third important theme. This especially revolved around the indicators to be included in the dashboard (ie, during stages 2 and 3). Here, interviewees appreciated the attention that was given to selecting indicators that, from the perspective of health professionals, would be most relevant in achieving improvements in the quality of care in that care pathway:

There is of course a difference between indicators, where the gynecologists, for example, want to count frequency of something and which they then pick from another list. That’s great, but that’s not helpful for frontline professionals. So we really looked at: what is good for frontline professionals and what is useful to them?

R11, nurse

While acknowledging the importance of selecting indicators that have relevance, quality and safety supervisors did focus on also steering toward the inclusion of process and outcome indicators (including clinical outcomes, patient-reported outcome measures, and patient-reported experience measures), for which it is known performance is lacking. This was done to ensure a wide variety of indicators are included and progress can be made in some areas where improvement is most beneficial:

There, there is of course, I wouldn’t call it secretly, but a bit of an invisible task for us to guide toward, to, if we know that at a certain department something is going on or you know that a certain department indictor from the inspectorate [national quality and safety inspectorate] has been below par for years.... Then I think I would find that I didn’t have a successful session if we finish it and that indicator is not present on the dashboard.

R2, quality and safety supervisor

In selecting indicators, proper definition and registration were frequently mentioned as key elements. Without a clear definition of what an indicator entails and how this should be registered, the dashboard was said to not have much-added value as performance cannot be accurately measured. As can also be seen in Table 2, interviewees stated that during the process it is important to create consensus on the definition of an indicator, as differences in interpretation can occur among health professionals. For example, “length of stay” can be considered by some as only the time spent at their department, whereas others consider it the total time spent in the hospital as a whole. Regarding registration, interviewees commented on differences that can occur as well, with some health professionals, for example, filling in indicator information in the free-text section of the electronic patient record instead of selecting a variable, which can then not be used for the dashboard.

Currently with EPD [electronic patient records] that’s difficult, with everyone putting it [indicator information] somewhere else, but that’s something to consider. That when you build something new, that you ensure that it’s unambiguous, because then you get the most clear data.... Then you don’t have that bad registration is bad performance, but then you have good registration with which you can do something.

R6, medical specialist

## Discussion

### Principal Findings

To our knowledge, this is the first study examining important factors for successfully developing quality and safety dashboards together with health professionals. We found that a 5-stage process can be used, whereby creating and maintaining professional involvement, unburdening, and focusing on relevancy for health professionals are important aspects to consider for successful development.

Our results show that when including health professionals in the development process, attention should be given to ensuring the inclusion of the various functions of a care pathway so as to create representativeness and a broad support base. Our research, therefore, extends earlier work where health professionals were not involved in the process [13,14,17-19]. While we find that including the various functions can be achieved rather easily, ensuring broad involvement can be more challenging. Here, tensions arise between not being able to include the entire workforce while still trying to find ways of engaging them in the process. In line with recent research, we find that having “innovative champions” (ie, staff with an interest in innovation, acting as linchpins) could be a means to achieve broad engagement whilst keeping the process manageable [34,35]. Here, it is important that these champions are aware of their role and consult with their peers regularly, giving them the possibility to provide input. A further challenging aspect regards maintaining involvement during the implementation stage, whereby daily tasks seemed to get in the way of frequent consultation of the dashboard. A greater focus on structural embeddedness during this stage, with time being created during team meetings to evaluate the dashboard, might be a means to overcome this issue, as shown by earlier studies [13,14]. Ultimately, frequent team evaluations of quality and safety dashboard performance could then become routine and be used to engage in continuous quality improvement.

We know from the literature that engaging health professionals in quality improvement initiatives, in our case, the development of quality and safety dashboards, can be challenging due to a lack of priority and perceived burden [36]. To increase the likelihood of engagement, clear communication of goals and health professionals’ benefits are among the suggestions made [36]. We furthermore found that good preparation, having structured and planned sessions in advance, and clear follow-up by quality and safety supervisors can be important attributes for good engagement in the development process, as the burden for health professionals is kept to a minimum. This is in line with the Deming System of Profound Knowledge, whereby the process is set up in such a way that it accommodates staff’s needs and requires as little effort as possible [37]. In order to have a smooth process and keep the burden on health professionals to a minimum, quality and safety employees are further advised to closely collaborate with the departments that are involved in designing the quality and safety dashboard and ensuring proper data infrastructure, such as the BI and IT departments.

When including health professionals in the development process, focusing on relevance was found to be important, and this seems to follow rather naturally through the inclusion of the various functions and the focus on unburdening. While in itself important, as it was deemed a positive aspect by interviewees, focusing on what health professionals consider important can also contribute to the greater goal of improving performance on quality and safety as it is an important prerequisite for organizational learning [38]. When aspects are measured that matter to health professionals, their willingness to engage in evaluations and aim for improvements is likely to increase [39]. However, considering the ultimate goal of quality and safety dashboards—to improve quality and safety performance—it might be beneficial to have a certain balance between selecting indicators that are merely relevant for health professionals and selecting those that, from a quality and safety perspective, are important in pursuing quality and safety improvements. Here we build on earlier research describing types of indicators that can be included in quality and safety dashboards [9,13,14,17-19], whereby we propose to seek this balance. Our results show that quality and safety supervisors aimed to do so by “invisibly” steering toward certain indicators where they deemed great progress could be made. In line with previous studies, we furthermore found that it is important that the definition and registration of indicators are clear to health professionals in order to have reliable data and thus be able to measure performance [25,26], which can be achieved by reaching a consensus on the definition and registration during the development process. A recent study by Khanbhai et al [23], however, showed that reaching a consensus might not always be necessary, as there can be instances where participants prefer qualitative data. They showed that for a patient experience feedback dashboard, participants valued being able to view patients’ comments, which was facilitated by augmenting the dashboard with free-text data [23].

### Conclusions and Practical Implications

Overall, our findings have important implications for practice, as they can be used by health care organizations seeking to develop quality and safety dashboards in collaboration with health professionals. Specifically, organizations can facilitate the process through 5 stages. The first stage revolves around familiarizing participants with the development process and the concept of dashboards. In the second stage, participants can brainstorm about potential indicators to be included. Next, through prioritizing, defining, and selecting indicators, participants can decide on the indicators to be incorporated into the dashboard. The fourth stage then focuses on how the selected indicators can be visualized. The fifth stage can be used for the implementation of the dashboard and to monitor its usage.

To enhance the success of the process, health organizations should take 3 main factors into account during the course of this process. First, our findings suggest that creating and maintaining the broad involvement of health professionals is an important prerequisite for a successful process (see Table 2). Here, a potential main barrier could be gaining engagement from peers not directly involved in the process, which could be resolved by selecting enthusiastic participants for the process who frequently report back to and consult with their peers. Moreover, maintaining involvement after initial implementation may be an issue, for which incorporating routine evaluations during team meetings might be a solution. A second aspect regards a focus on unburdening by those guiding the sessions, whereby the process is organized such that it has a little additional burden for participants. Our results suggest time management might become an issue here, causing a lack of progress. A possible solution would be to foreseeably schedule the different sessions and incorporate a certain degree of strictness. A further issue could be collaborating with the IT and BI departments, who might not be aware of what is expected of them. Expectation-setting and frequent communication could thus be important. Lastly, a key element of a successful development process entails focusing on including indicators in the dashboard that have value for health professionals. Here, the main barrier regards a lack of clarity in defining and registering indicators, resulting in unreliable data. During the development process, consensus should thus be reached on how indicators should be defined and registered.

To conclude, both in research and practice, there is growing interest in involving health professionals in the development of quality and safety dashboards. Our findings show that this can be facilitated through a 5-stage process, whereby success can be enhanced by taking into account 3 key factors. Overall, this could increase the likelihood that the dashboards will be used in practice.

### Limitations

Our study was of a retrospective nature and requires additional research to further parse out the findings. While 150 pages of written documents ensured a certain degree of reliability, the data retrieved through the interviews might be subject to recall bias. Secondly, the findings are based on a limited sample size. Although the literature suggests our sample size is sufficient for reaching data saturation and we included participants from 2 care pathways to broaden the scope, insights from other studies conducted in other countries could increase generalizability. As a third limitation, our findings provide insight into factors that need to be considered when including health professionals in the development process of quality and safety dashboards but provide less insight into the effect of such involvement on the use of the dashboard in the long term and improvements in quality and safety performance. Future longitudinal studies are needed to examine whether, as suggested by the literature, the inclusion of health professionals in the development process indeed increases the use of the quality and safety dashboard by care pathway staff and leads to improvements in quality and safety performance.

## Appendix

Multimedia Appendix 1 Interview guides.

Multimedia Appendix 2 Coding tree.

## Abbreviations

BI: business intelligence
COREQ: Consolidated Criteria for Reporting Qualitative Research
IT: information technology

## Data Availability

The data sets generated during and/or analyzed during this study are available in the Health Services Research repository of Maastricht University [40].
