# Supplementary material for: Involving Health Professionals in the Development of Quality and Safety Dashboards: Qualitative Study
Source: J Med Internet Res. 2023 Jun 12;25:e42649. doi: 10.2196/42649 (PMC10337379; doi:10.2196/42649)
Supplement: Multimedia Appendix 1 [file jmir_v25i1e42649_app1.docx]

**Multimedia Appendix 1: Interview guides**

**Interview guide Quality and Safety supervisors**

*Topics*

- Preparations
- Content of the sessions
- What went well/what could be improved. Lessons learned

**Main question**

What does the process of developing quality and safety dashboards together with health professionals entail, and what are important aspects?

Introduction

Personal introduction, providing research background, study aim. Answering potential questions of interviewee.

- Could you tell something about your function and work experience at the hospital?
- When did the initial ideas arise of developing quality and safety dashboards together with health professionals?

Preparations

- What preparations were made before the process was started?
  - How was it decided that the process should consist of five stages?
  - How were the sessions created? (e.g. based on literature, earlier experiences)
  - How was it decided who should be involved in the process?
  - What are point to consider when preparing for the process?

Sessions

- Session 0. Kick-off development quality and safety dashboard
  - What did this session revolve around?
  - What is important to consider in order to ensure a successful session?
  - Are there aspects that could gone better, or on the other hand things that went really well?
- Session 1. Inventory
  - What did this session revolve around?
  - What is important to consider in order to ensure a successful session?
  - Are there aspects that could gone better, or on the other hand things that went really well?
- Session 2. Prioritize and define
  - What did this session revolve around?
  - What is important to consider in order to ensure a successful session?
  - Are there aspects that could gone better, or on the other hand things that went really well?
- Session 3. Visualize
  - What did this session revolve around?
  - What is important to consider in order to ensure a successful session?
  - Are there aspects that could gone better, or on the other hand things that went really well?
- Session 4. Implement
  - What did this session revolve around?
  - What is important to consider in order to ensure a successful session?
  - Are there aspects that could gone better, or on the other hand things that went really well?

Additional points

It is a diverse group of people that is involved in the process (e.g. medical specialists, nurses). Do you see that this has an influence on the process? Are there points to consider?

Looking at the process as a whole, what are important elements to consider in order to ensure that the process goes well/succeeds?

If the dashboard would have been development in a different way, do you think it would have gone better/worse?

Are there things you would do differently next time, why?

**Interview guide participants**

*Topics*

- Content of the sessions
- What went well/what could be improved. Lessons learned

**Main question**

What does the process of developing quality and safety dashboards together with health professionals entail, and what are important aspects?

Introduction

Personal introduction, providing research background, study aim. Answering potential questions of interviewee.

- Could you tell something about your function and work experience at the hospital?
- When did you become involved in the process?
  - Was this on time/too soon/too late, why?
  - What were your initial thoughts about quality and safety dashboards and the development process?

Sessions

- Session 0. Kick-off development quality and safety dashboard
  - What is important to consider in order to ensure a successful session?
  - Are there aspects that could gone better, or on the other hand things that went really well?
- Session 1. Inventory
  - What is important to consider in order to ensure a successful session?
  - Are there aspects that could gone better, or on the other hand things that went really well?
- Session 2. Prioritize and define
  - What is important to consider in order to ensure a successful session?
  - Are there aspects that could gone better, or on the other hand things that went really well?
- Session 3. Visualize
  - What is important to consider in order to ensure a successful session?
  - Are there aspects that could gone better, or on the other hand things that went really well?
- Session 4. Implement
  - What is important to consider in order to ensure a successful session?
  - Are there aspects that could gone better, or on the other hand things that went really well?

Additional points

It is a diverse group of people that is involved in the process (e.g. medical specialists, nurses). Do you see that this has an influence on the process? Are there points to consider?

Looking at the process as a whole, what are important elements to consider in order to ensure that the process goes well/succeeds?

If the dashboard would have been development in a different way, do you think it would have gone better/worse?

Are there things you would do differently next time, why?
