# Supplementary material for: Involving Health Professionals in the Development of Quality and Safety Dashboards: Qualitative Study
Source: J Med Internet Res. 2023 Jun 12;25:e42649. doi: 10.2196/42649 (PMC10337379; doi:10.2196/42649)
Supplement: Multimedia Appendix 2 [file jmir_v25i1e42649_app2.docx]

**Multimedia Appendix 2: Coding tree**

| **First order codes** | **Second order codes** | **Third order codes** |
| --- | --- | --- |
| Representation of different functions  Peer engagement  Support base  Affinity with quality and safety | Creating broad involvement | Creating and maintaining broad involvement |
| Lack of frequent consultation  Day-to-day routines  Progress tracking during team meetings  Structural feedback to whole department  Positive feedback | Maintaining broad involvement |  |
| Clear goals  Proper planning  Smooth transition  Time efficient  Low effort | Structure | Unburdening |
| Follow-up  Task clarity  Collaboration BI  Collaboration IT  Guiding the process | Good guidance |  |
| Indicators with value  Reflect on value indicators with peers  Diverse KPI’s  Steering | Value indicators | Focussing on relevance for health professionals |
| Definition important  Proper registration  Accurate measurement  Free-text data | Definition and registration |  |
